# Prevalence of colonisation with vancomycin-resistant enterococci on admission - a cross-sectional study in 6 German university hospitals, 2014

**DOI:** 10.1186/2047-2994-4-S1-P198

**Published:** 2015-06-16

**Authors:** M Wiese-Possselt, J Zweígner, AM Rohde, F Schwab, W Kern, H Seifert, P Gastmeier, the DZIF-ATHOS Study Group

**Affiliations:** 1 Institute of Hospital Hygiene, Charité, Berlin, Germany; 2 University Hospital Cologne, Germany; 3 Charité, Germany; 4 University Hospital Freiburg, Germany

## Introduction

The survey presented here is part of the multicenter study ATHOS (antibiotic therapy optimisation study) on prevalence and incidence of nosocomial carriage of multi-drug resistant organisms (MDROs) in Germany.

## Objectives

The aim of this survey was to assess the prevalence of rectal colonisation with VRE in patients on hospital admission. Additionally, we performed a risk factor analysis for VRE carriage.

## Methods

We recruited adult patients within 72 h after admission to non-intensive care units in six German university hospitals to obtain rectal swabs that were screened for VRE. Each patient was asked to answer a short questionnaire on potential risk factors for colonisation with MDROs, such as sex, age, antibiotic therapy in the last 6 months, previous colonisation with any MDRO, travel abroad in the last 6 months, stay in a nursing home or hospitalisation in the last 6 months. Univariable and multivariable risk factor analyses were performed to identify those factors which were associated with VRE colonisation.

## Results

In 2014, 4372 patients were included in the admission survey. Overall, 35 patients were colonised with VRE (admission prevalence of 0.8%). The following factors were associated significantly with VRE carriage: centre (OR = 3.61, CI 95% 3.05-4.27, p<0.001) , previous MDRO colonisation (OR = 3.23, CI 95% 1.47-7.11, p = 0.004), antibiotic use (OR = 2.91, CI 95% 1.47-5.74, p = 0.002), stay in a nursing home (OR = 3.19, CI 95% 1.87-5.46, p<0.001), and hospitalisation (OR = 3.19, CI 95% 2.06-5.42, p = 0.000). Interestingly, travel abroad had a protective effect on VRE carriage (OR = 0.51, CI 95% 0.29-0.91, p<0.001).

## Conclusion

This admission survey in Germany revealed a moderate colonisation rate of 0.8%, whereat prevalence differed significantly by centre. Well-known risk factors for VRE carriage were identified: previous colonisation with any MDRO, antibiotic use in the last 6 months, and admission to nursing homes or hospitals in the last 6 months. We observed that not travelling abroad was significantly associated with VRE carriage which we cannot explain. Thus, we cannot exclude a confounding factor we have not considered so far and should be addressed in further studies.

## Disclosure of interest

None declared.

